# Surgical treatment of peri-implantitis

**DOI:** 10.1038/s41415-024-7405-9

**Published:** 2024-05-24

**Authors:** Mario Roccuzzo, Davide Mirra, Andrea Roccuzzo

**Affiliations:** 41415122581001 Private Practice, Torino, Italy; Division of Maxillofacial Surgery, University of Torino, Italy; Department of Periodontics and Oral Medicine, University of Michigan, Ann Arbor, Michigan, USA; 41415122581002 Private Practice, Torino, Italy; 41415122581003 Department of Periodontology, School of Dental Medicine, University of Bern, Bern, Switzerland; Department of Oral and Maxillofacial Surgery, Copenhagen University Hospital (Rigshospitalet), Copenhagen, Denmark

## Abstract

As utilisation of dental implants continues to rise, so does the incidence of biological complications. When peri-implantitis has already caused extensive bone resorption, the dentist faces the dilemma of which therapy is the most appropriate to maintain the implant. Since non-surgical approaches of peri-implantitis have shown limited effectiveness, the present paper describes different surgical treatment modalities, underlining their indications and limitations. The primary goal in the management of peri-implantitis is to decontaminate the surface of the infected implant and to eliminate deep peri-implant pockets. For this purpose, access flap debridement, with or without resective procedures, has shown to be effective in a large number of cases. These surgical treatments, however, may be linked to post-operative recession of the mucosal margin. In addition to disease resolution, reconstructive approaches also seek to regenerate the bone defect and to achieve re-osseointegration.

## Key points

- To provide an overview on the different available treatment options.
- To summarise the clinical scenarios where reconstructive procedures of peri-implantitis are indicated.
- To provide an overview of the benefits of performing reconstructive procedures of peri-implantitis.

## Indications for surgical management of peri-implantitis

Effective management of peri-implantitis aims to decontaminate the infected implant surface and reduce the peri-implant pocket depth to ≤5 mm.^1^ To achieve this, various strategies have been proposed, drawing from periodontal therapy and encompassing both non-surgical and surgical interventions.^2^

Despite numerous attempts to treat peri-implantitis through non-surgical means, such as mechanical debridement or flapless approaches, often coupled with adjunctive measures like antibiotics or laser therapy, clinicians have observed limited improvements in clinical parameters, such as peri-implant probing pocket depth (PPD) reduction and bleeding on probing (BoP).^3^^,^^4^ The challenge lies in gaining proper access to implant surfaces for thorough decontamination and biofilm removal, particularly in cases with deep peri-implant pockets and diverse implant surface designs.

Accordingly, the European Federation of Periodontology (EFP) S3 clinical guidelines recommend using non-surgical protocols to establish healthier peri-implant soft tissue conditions before considering adjunctive surgical therapy.^5^ Surgical intervention necessitates a meticulous assessment of patient and implant factors influencing early healing and long-term outcomes.

The surgical procedure typically involves raising a full-thickness flap to access the contaminated implant surface, followed by degranulation of soft tissue defects and thorough peri-implant surface decontamination. Various hand- and power-driven devices have been proposed over the years to maximise biofilm removal while preserving the integrity of the titanium implant surface. However, no single device has demonstrated superiority in peri-implant surface decontamination.^5^^,^^6^^,^^7^

Recent advancements include a novel electrolytic cleaning device that applies a voltage to the implant fixture while delivering a sodium formate solution directly onto the titanium implant surface.^8^ Despite promising pre-clinical and short-term clinical results, routine use of this device is not currently recommended.

In light of available evidence, a dual approach combining mechanical and chemical decontamination is advised before evaluating the configuration of peri-implant bone defects.^5^ From a clinical standpoint, two major treatment modalities emerge:

- Access flap procedures, possibly combined with resective techniques or implantoplasty, and
- Reconstructive procedures aimed at restoring lost peri-implant bone using bone substitutes.^9^

## Access flap debridement without resective procedures

Access flap debridement (AFD) involves raising a mucoperiosteal flap and subsequently removing inflammatory tissue to access the contaminated implant surface. Following granulation tissue removal, the implant surface is decontaminated using mechanical, chemical, and potentially other adjunctive methods such as photodynamic therapy or laser treatment. The flap is then repositioned and sutured in place.

Studies have demonstrated the efficacy of open flap debridement without resective procedures, with favourable survival rates and moderate success rates (eg PPD ≤5 mm, absence of bleeding/suppuration on probing, and no progressive bone loss) observed up to five years post-treatment.^10^^,^^11^ However, despite initial success, maintaining good, long-term results poses challenges. Studies have reported that sustained disease resolution could only be achieved in 53% of implants and 63% of patients following AFD, even when coupled with systemic antimicrobial therapy.^12^

## Access flap debridement with resective procedures

In a five-year study analysing a resective approach involving bone recontouring and systemic antimicrobial therapy, 54% of implants achieved successful outcomes defined as disease resolution.^13^ However, 44% of implants experienced disease recurrence or progression, leading to the removal of 27 implants. Notably, a statistically significant correlation was observed between residual peri-implant PPD ≥6 mm at one-year follow-up and reduced marginal bone levels, indicating a higher risk of recurrence or progression of peri-implantitis. Additionally, implants with modified surfaces exhibited a greater risk of disease progression compared to those with non-modified surfaces.

A long-term retrospective cohort study spanning up to 11 years has demonstrated favourable clinical and radiographic outcomes following AFD combined with osseous recontouring.^14^ The study emphasised the influence of implant surface type, with turned surfaces yielding better results than modified rough surfaces.^13^

The impact of implant surface features on implant survival and success rates following surgical treatment of peri-implantitis has been previously highlighted by various authors.^15^^,^^16^ Bone grafting with deproteinised bovine bone mineral (DBBM) and 10% collagen showed varying implant survival rates with sand-blasted and acid-etched implants exhibiting an 80% survival rate compared to 55% for titanium plasma-sprayed implants over a 7-10-year follow-up period.

Implantoplasty involves smoothing supracrestal implant threads and exposed rough surfaces to aid biofilm removal and minimise its adherence during the maintenance phase. However, current evidence regarding the advantages of implantoplasty over other decontamination methods in the surgical treatment of peri-implantitis is inconclusive.

Comparative studies have evaluated the efficacy of implantoplasty against alternative decontamination with air-polishing with glycine powder.^17^ Clinical evaluations at three and six months showed similar outcomes in terms of PPD and BoP, suggesting comparable effectiveness between implantoplasty and glycine air-polishing.

However, concerns regarding implantoplasty, including residual titanium particles in peri-implant soft tissues or implant fracture, warrant further investigation.^18^ It is advisable to exercise caution when performing implantoplasty with narrow-diameter implants that may be become more prone to fracture.^19^

Clinically, it is essential to consider the potential increase in post-operative peri-implant mid-buccal soft tissue recession associated with implantoplasty compared to access flap and reconstructive procedures, particularly in cases where aesthetics may be compromised.^20^^,^^21^

## Indications and efficacy of reconstructive approaches

After the removal of the granulation tissue and the decontamination of the implant surface, surgical reconstruction is desirable, as it has the potential to restore the anatomy of the lost tissues, achieve re-osseointegration, and limit peri-implant soft-tissue recession. Systematic reviews have reported on the outcomes of such reconstructive procedures with mixed results. The wide variation of results may be attributed to the heterogeneity of the studies, with regards to the severity of the disease, and the differences in the surgical techniques employed. The consensus report of Group 4 of the 15th European Workshop on Periodontology on Bone Regeneration indicated which patient-related and site-related factors clinicians should consider when recommending reconstructive procedures in the surgical therapy of peri-implantitis.^22^

As part of the detailed planning process for reconstructive approaches in the treatment of peri-implantitis, clinicians should aim to meet the following patient-related conditions:

- Patient willing to undergo the intervention and effectively participate in a tailored supportive care peri-implant programme
- Realistic patient expectations
- Low full-mouth plaque scores (<20%)
- Low full-mouth bleeding scores (<20%)
- Smoking<10 cigarettes/day
- No medical contraindications for surgical/reconstructive intervention.

In addition to these patient-related factors, clinicians should also assess the following site-related factors:

- The depth of the intrabony defect >3 mm
- The defect configuration: ideally, an isolated three- or four-wall-contained defect
- Presence of a band of peri-implant keratinised mucosa.

After consideration of the most recent literature reports on the topic, it is clear that the vast majority of studies have not taken all these factors into consideration, and this creates confusion among the clinicians regarding when and where a reconstructive approach should be indicated.

The main objective of the XVIII European Workshop on Periodontology was to summarise the evidence-based recommendations for individual interventions used in the management of peri-implant diseases, based on the best available evidence and/or expert consensus. For the guideline development process on the efficacy of bone reconstructive therapies in the management of peri-implantitis defects, a systematic review was conducted by Donos and co-workers.^23^ Based on the meta-analysis, it was concluded that ‘both access flap and reconstructive surgery can significantly improve peri-implant clinical parameters at 12 months of follow-up, with reconstructive surgeries leading to improved radiographic outcomes'. Therefore, since reconstructive surgery does not seem to offer significant improvements in peri-implant clinical parameters as compared to access flap approaches, the clinician may be inclined to consider only the less complex approach (ie access flap surgery). It must be considered, however, that various methods of reconstructive surgeries were considered in the workshop: amelogenin; DBBM or DBBM graft with 10% collagen either alone or with the use of a collagen membrane; titanium granules; and beta-tricalcium phosphate graft with prolonged-release local doxycycline. Meta-analysis could only be performed to assess PPD changes between baseline and 12 months of follow-up for four studies which employed a bone graft associated or not with a barrier membrane, while studies employing titanium granules or bioactive factors alone were not included. 

Moreover, it was not possible to establish a hierarchy of efficacy among the different biomaterials employed for reconstructive surgery. This is not to say that currently used grafts are not effective. In particular, DBBM with 10% collagen alone has received much positive attention in the last years by several authors.^15^^,^^16^^,^^21^^,^^24^^,^^25^^,^^26^^,^^27^^,^^28^ Furthermore, some studies have reported that the use of a barrier membrane might enhance the risk of early complications, primarily soft-tissue dehiscence and exposure of the membrane/graft.^29^^,^^30^

It was beyond the remit of the review to provide indications related to the impact of the flap design and surgical management on the treatment outcome. However, these characteristics may play a crucial role when deciding on the surgical reconstructive approach. Indeed, it must be reaffirmed that reconstructive treatment of peri-implantitis defects is much more than simply adding a graft to an access flap, as has often been investigated during randomised controlled trials to date.

In consideration of these limitations, there is not presently a strong evidence-based approach for reconstructive surgery in peri-implantitis defects. The clinical management of such cases tends to be based on ‘expert opinion' and is taken from experience in the regenerative treatment of the periodontal defects. A fundamental goal of such surgical approaches is to limit the postsurgical recession of the soft tissues.

For the regenerative treatment of periodontal defects, several surgical techniques have been developed to optimise primary closure as well as to minimise surgical trauma in intraosseous defects. The basic principle consists of the elevation of a single flap (ie on the buccal or palatal/lingual aspect only, depending on the main extension of the defect) to access the defect, leaving at least one interproximal papilla intact.^31^

Another important aspect is the formation of an early and long-standing effective barrier around the collar of the implant, capable of biologically protecting the peri-implant structures.

In absence of clear evidence-based surgical guidelines, some general concepts should be kept in mind for the reconstructive therapy of peri-implantitis defects:

- Deep and narrow defects are more favourable for the stabilisation of the graft and the consequent formation of new bone
- The extension of the full-thickness flap should be planned carefully, balancing the aim to minimise the invasiveness of the procedure and the need to have access to the bottom of the defect
- The regenerative biomaterial should be selected on the basis of proper scientific validation and applied without overfilling the defect
- The graft should be inserted only after complete elimination of the granulation tissue and the decontamination of the implant surface
- In case of insufficient keratinised mucosa width, a connective tissue graft should be trimmed and adapted over the entire defect so as to cover 2-3 mm of the surrounding alveolar bone to ensure stability of the graft material. Should the defect be circumferential in an area with no keratinised mucosa, a large connective tissue graft could be punched, by means of a circular blade, and adapted circumferentially around the defect
- Patients should be instructed on how to protect, care for, and keep clean the surgical site so as to favour primary healing of the surgical wound.

**Fig. 1:**
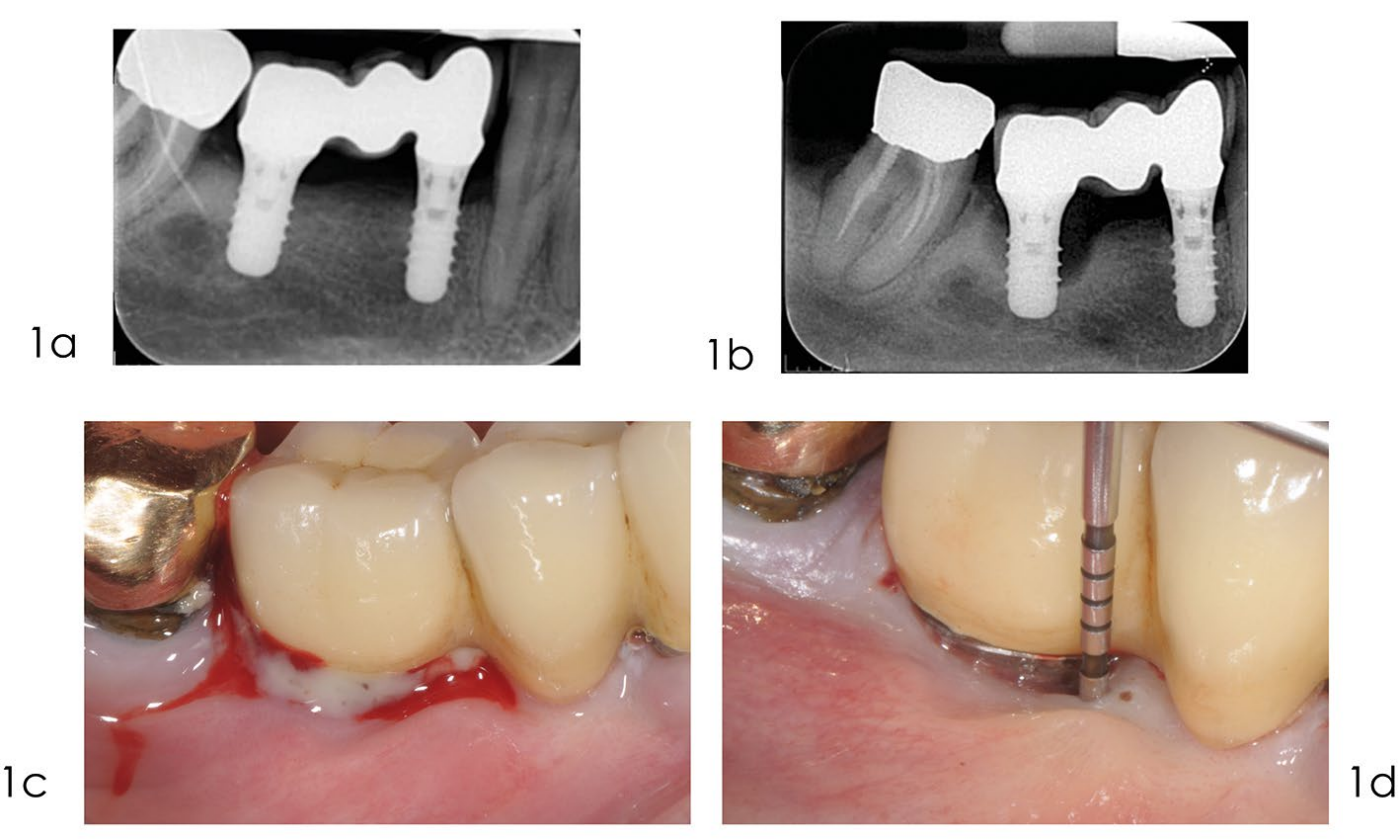
Reconstructive treatment of peri-implantitis: diagnosis. a) Radiograph taken in September 2010, three years after delivery of the prosthesis, reveals optimal peri-implant bone levels. b) Radiograph taken in December 2014 depicts advanced marginal bone loss at implant 4.6. c) Bleeding on gentle probing and/or suppuration are the main clinical characteristics of mucositis and peri-implantitis. d) Peri-implantitis sites exhibit clinical signs of inflammation, increased probing depths, in addition to radiographic bone loss compared to previous examinations. There is some evidence linking peri-implantitis to the lack of keratinised mucosa

**Fig. 2:**
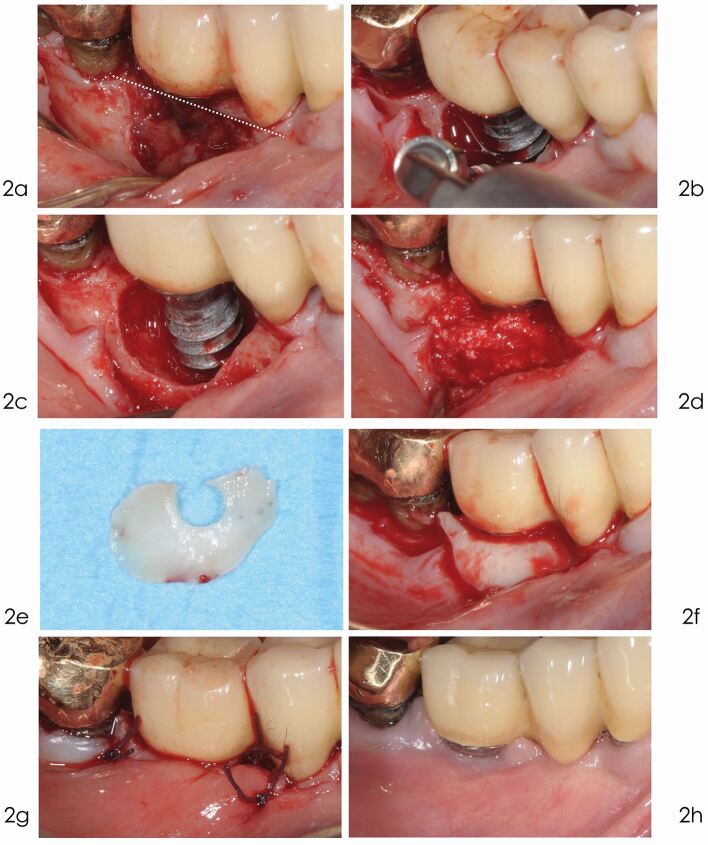
Reconstructive treatment. a) A linear crestal incision was performed leaving the mesial papilla into place in order to facilitate the stabilisation of the flap over the grafted defect. b) The exposed implant surface was thoroughly cleaned using an ultrasonic device with a Teflon-coated tip, under continuous saline irrigation. c) After the removal of granulation tissue the implant surface is decontaminated with ethylenediaminetetraacetic acid (EDTA) 24% and chlorhexidine 1% gel. d) Deproteinised bovine bone mineral with 10% collagen is applied in the infrabony defect. e) A connective tissue graft is taken from the maxillary tuberosity and U-shaped. f) The connective tissue graft is adapted around the collar of the implant and over the entire defect to ensure stability of the graft. g) 4/0 Vycril suture of the flap ensures an optimal not-submerged healing. h) Optimal one-year healing. No signs of inflammation. A keratinised tissue band is now visible around the implant

**Fig. 3:**
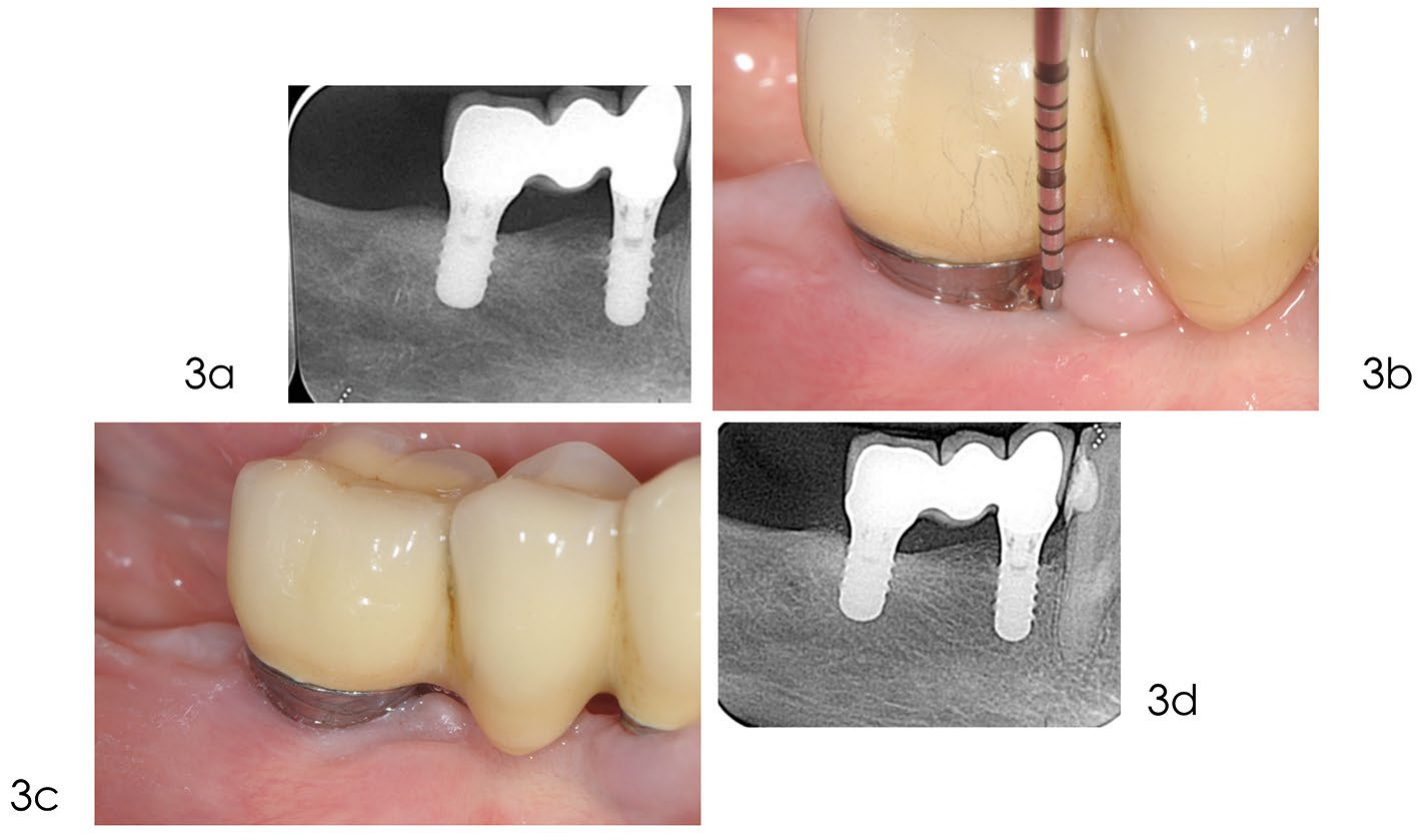
Follow-up (supportive periodontal care). a) Radiograph taken in May 2019 reveals complete bone fill of the defect. b) After the surgical treatment, the patient was asked to follow an individualised supportive care program including oral hygiene measures, biofilm removal and monitoring risk indicators. c) Clinical picture in November 2021 demonstrates healthy peri-implant tissues. The probing reveals shallow pocket and the absence of bleeding. d) Radiograph taken in March 2023, 16 years after implant placement, shows optimal interproximal bone levels

The case presented in Figure 1, Figure 2 and Figure 3 illustrates a typical successful treatment of a severe peri-implantitis defect.

With these concepts in mind, it is reasonable to state that intrabony peri-implantitis defects, around properly placed implants, can be successfully treated by means of reconstructive surgery in a high percentage of cases.

## Conclusions

The primary goal of the treatment of peri-implantitis is to eliminate the infection and to arrest the progression of peri-implant bone loss.

Unlike for periodontitis, non-surgical treatment is usually inadequate to treat peri-implantitis, particularly in advanced cases with deep bone defects. Nevertheless, non-surgical treatment usually represents the first step of care, as it creates better peri-implant conditions before surgical intervention.

Regardless of the surgical treatment approach selected, adequate plaque control is fundamental to achieving treatment success.

Access flap surgery may be linked to post-operative recession of the mucosal margin and consequent soft tissue dehiscences, which may create aesthetic problems.

Reconstructive therapies attempt to recreate ideal hard and soft-tissue conditions, in order to facilitate the long-term maintenance and to preserve aesthetics.

The reconstructive treatment of peri-implantitis includes specific flap designs with the objectives to minimise surgical trauma, maintain the interproximal tissue and maintain the level of the peri-implant mucosal margin.

Flap design may play a key role in enhancing the outcomes in reconstructive therapy of peri-implantitis defects and should be dictated by the defect configuration (shape, number of walls, depth), defect severity, implant type, implant position and peri-implant soft tissue characteristics, and all of these factors make randomised controlled trials difficult to conduct.

**Fig. 4:**
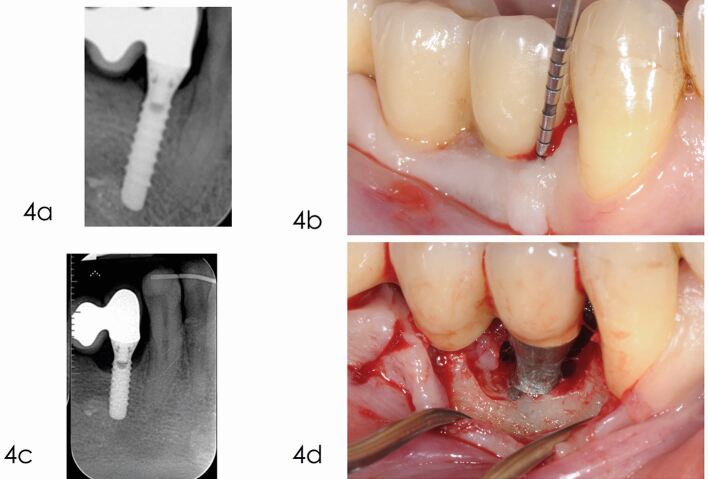
Access flap debridement. a) Radiograph taken one year after delivery of the prosthesis reveals optimal peri-implant bone levels. b) Bleeding on gentle probing and pocket depth of 6 mm, five years after implant placement. c) Radiograph taken in December 2014 depicts marginal bone loss at implant 4.4. d) The elevation of a full thickness flap revealed the bone loss at the level of the second thread. Due to the thin bone crest with no infrabony component, a reconstructive approach was not selected, but an open flap debridement was preferred

**Fig. 5:**
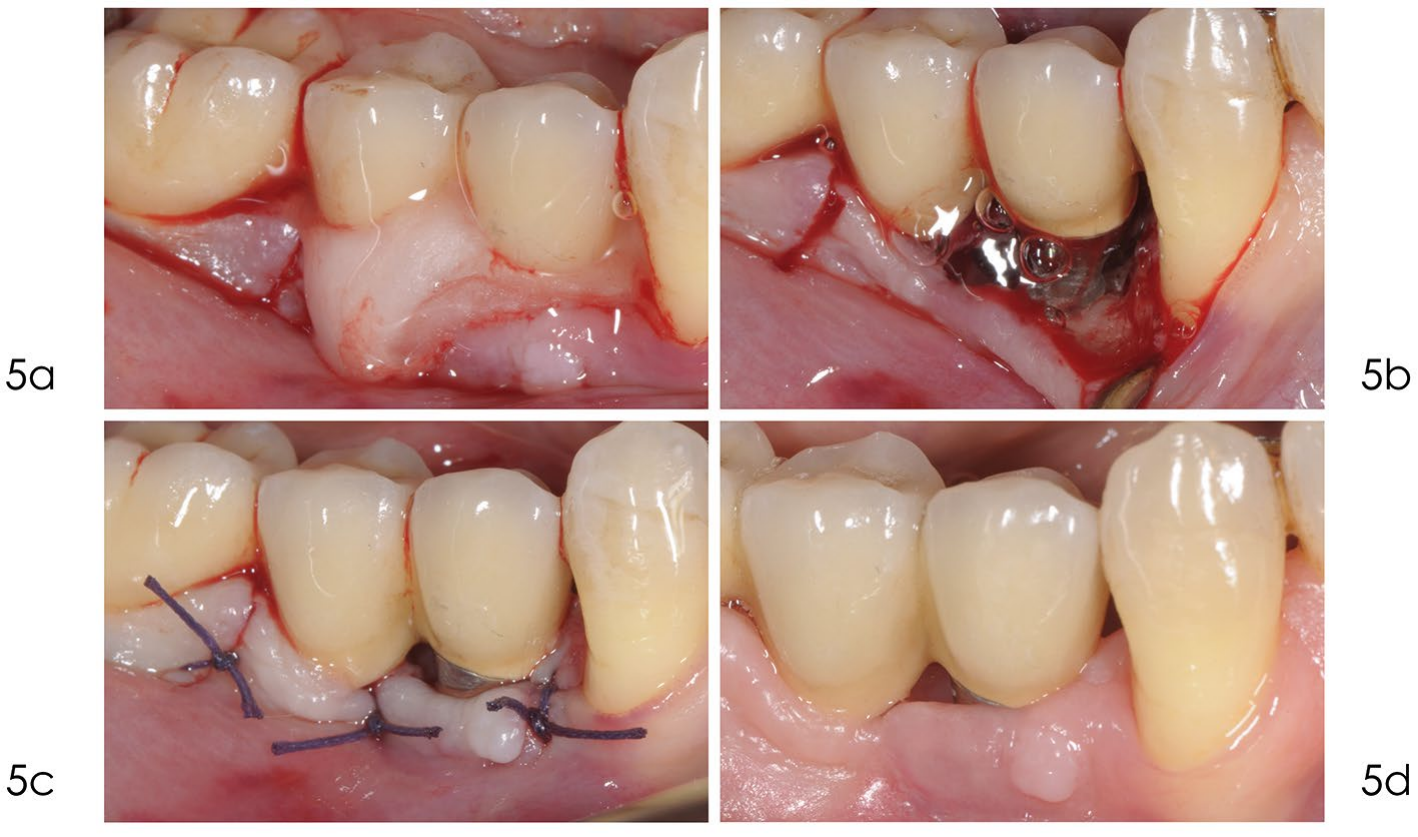
a) After the removal of granulation tissue, the implant surface was decontaminated with EDTA 24% for two minutes. b) After saline irrigation, chlorhexidine 1% gel was applied for two minutes. c) 4/0 Vycril suture of the flap ensures an optimal not-submerged healing. d) Healing proceed with no complications

**Fig. 6:**
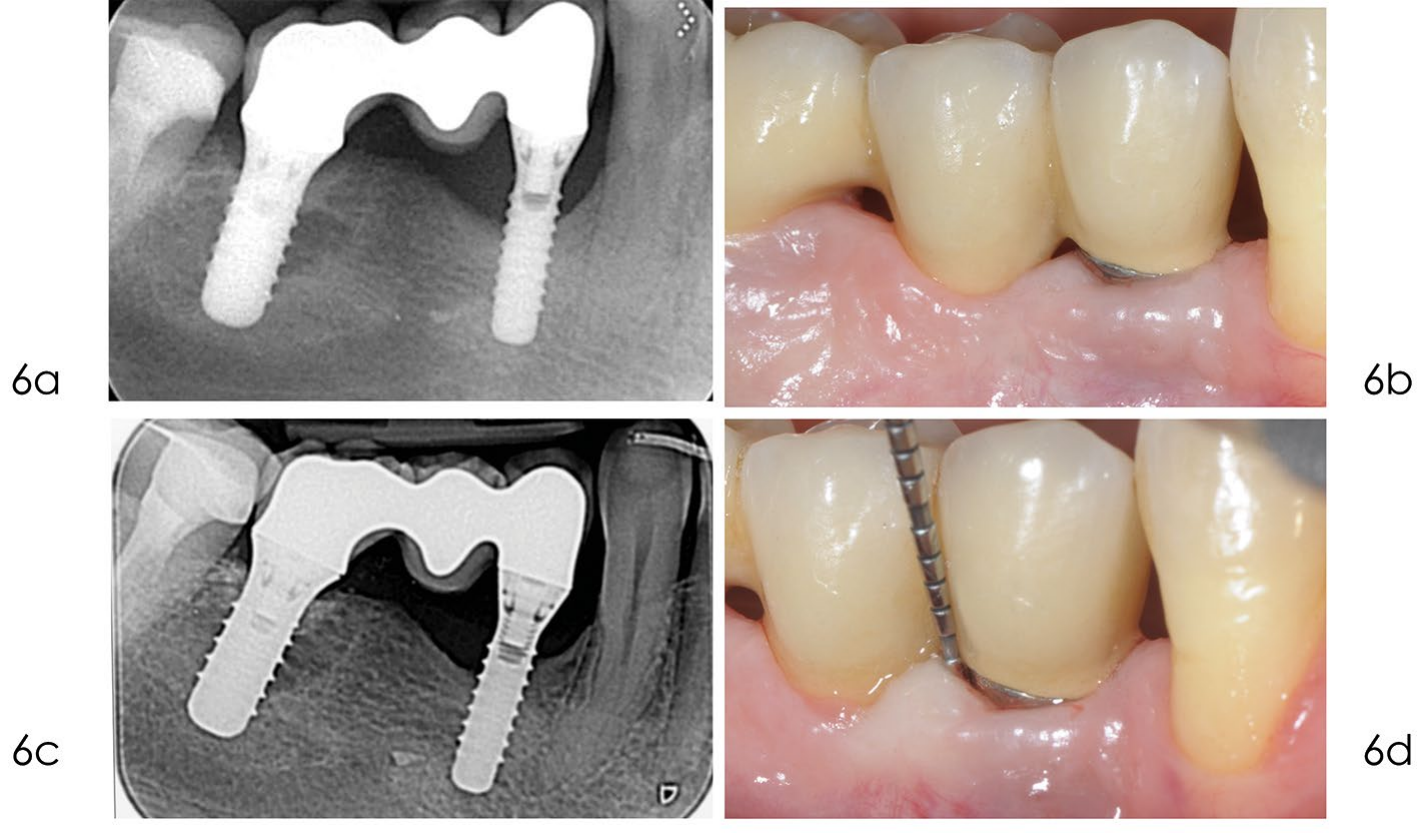
a) Radiograph taken in September 2020, ten years after implant placement, shows stable bone defect. b) Clinical picture in September 2020 shows peri-implant soft tissues free from inflammation. c) Radiograph taken in January 2024 confirm the absence of additional bone loss distally, and minimal improvement on the mesial aspect. d) Clinical picture in January 2024 demonstrates a minimal soft tissue recession, 14 years after placement. The probing reveals stable, even if not ideal, pocket depth

The use of a minimally invasive approach should be encouraged in order to minimise trauma to the tissues and must be weighed against the requirement to obtain adequate access to the entire bone defect (see Figures 4, 5 and 6).
